# General practice variation in peptic ulcer prophylaxis: a nationwide register-based study

**DOI:** 10.1080/02813432.2024.2396871

**Published:** 2024-08-30

**Authors:** Peter Fentz Haastrup, Jane Møller Hansen, Jens Søndergaard, Dorte Ejg Jarbøl

**Affiliations:** a Research Unit of General Practice, Department of Public Health, University of Southern Denmark, Odense, Denmark; b Department of Medical Gastroenterology, Odense University Hospital, Odense, Denmark

**Keywords:** Peptic ulcer prophylaxis, gastroenterology, proton pump inhibitors, general practice variation, phamacology

## Abstract

**Background:**

Incidence of peptic ulcer bleeding can be substantially reduced by prophylactic use of proton pump inhibitors (PPIs) in patients at risk, but use of PPI varies among risk patients, and substantial under-prescribing may exist. The variation in prophylactic prescribing among general practices remains unknown.

**Methods:**

A nationwide register-based cross-sectional study analyzing the proportion of patients at risk of ulcer bleeding receiving PPI treatment within Danish general practices. Using logistic regression, we analyze associations between general practice characteristics and prophylactic treatment among patients at risk of ulcer bleeding listed with the general practice.

**Results:**

In most general practices, less than 40% of the patients at increased risk of ulcer bleeding were covered by PPI. Geographical variation was present, where practice location outside the capital area was associated with higher odds of PPI coverage among their risk patients. Partnership practices with GPs with a mean age ≥65 years or with only female GPs were associated with higher odds of providing prophylaxis among their risk patients compared to practices with a mean GP age <45 years or with only male GPs. Similar associations were not found for single-handed practices.

**Conclusions:**

A significant under-prescribing of ulcer prophylaxis is common across all general practice characteristics, and only few associations with practice characteristics were present. Most efforts to rationalize PPI prescribing have aimed at reducing overprescribing but the findings point to under-prescribing as a problem as well. Development of new methods to assist GPs in identifying individuals at risk of ulcer complications is needed.

## Introduction

Peptic ulcer disease is a serious condition associated with substantial morbidity and mortality if complicated by hemorrhage [1]. Despite a decrease in upper gastrointestinal bleeding, it remains a common and potentially life-threatening event [2]. The most recent Danish evaluation shows an incidence of bleeding peptic ulcer of 0.57 per 1000 person years [3]. Risk factors for peptic ulcer bleeding comprise age, sex, concomitant use of non-steroidal anti-inflammatory drugs (NSAIDs), anticoagulants, antiplatelets, etc. [4]. Especially concomitant use of the antiplatelet subtype adenosine diphosphate (ADP) receptor inhibitors or anticoagulants or a history of previous ulcer increases the risk of peptic ulcer complication substantially [5]. Proton pump inhibitors (PPIs) are effective as gastroprotective agents and approximately halves the risk of peptic ulcer in patients at risk [4]. Danish general practice guidelines suggest prescribing prophylactic PPI to patients with indicated ulcerogenic medication (NSAID and/or low-dose aspirin) and previous ulcer complication or risk factors for ulcer complication such as age >65 years, substantial comorbidity, concomitant use of anticoagulants, antiplatelets, etc. [6]. While most research has addressed potential overuse of PPIs [7] and the possible risks of long-term PPI use [8], studies have also shown that PPIs are underused in patients at increased risk of peptic ulcer complications [9,10]. Several factors might account for the lack of concomitant prophylactic treatment. Patient factors in terms of medication use, age, sex and comorbidity are demonstrated to be associated with PPI prophylaxis among patients at increased risk of peptic ulcer bleeding [11]. Other patient-related factors such as non-adherence, polypharmacy and comorbidity could play an important role too, but physician-related factors may be of importance as well. Most prescriptions for PPI are issued by GPs [12,13]; hence, physician variation in PPI prescribing is relevant to study among GPs. Diagnosis and management of patients with chronic disease and adherence to guidelines are associated with general practice characteristics, where group practices and practices with lower workload have a higher score in certain performance indicators [12,14–16]. Further, we know that younger GPs and GPs who are women are less likely to initiate long-term prescribing of PPIs [12]. However, we do not know if patients listed with GPs with the abovementioned characteristics are less likely to receive PPI if being at increased risk of ulcer or how other general practice characteristics may be associated with insufficient peptic ulcer prophylaxis. Identification of practice characteristics associated with prescribing PPI in patients at increased risk of peptic ulcer bleeding may assist in developing targeted interventions to increase prophylactic prescribing of PPI and thereby decrease incidence of avoidable peptic ulcers. Hence, the aim of this study was to analyze associations between general practice characteristics and PPI prophylaxis in patients at increased risk of peptic ulcer bleeding.

## Methods

### Design and setting

This study is register-based and comprises the entire Danish population of approximately 5.8 million citizens and all general practices in Denmark (approximately 1700 practices). Most Danes are registered with a GP performing initial diagnostics and treatment with the possibility to refer patients for specialist investigations and treatment when necessary. All health care services are free of charge (tax-funded) with equal access for all citizens [17]. The average list size of a Danish GP is approximately 1600 patients [17].

**Figure 1.**
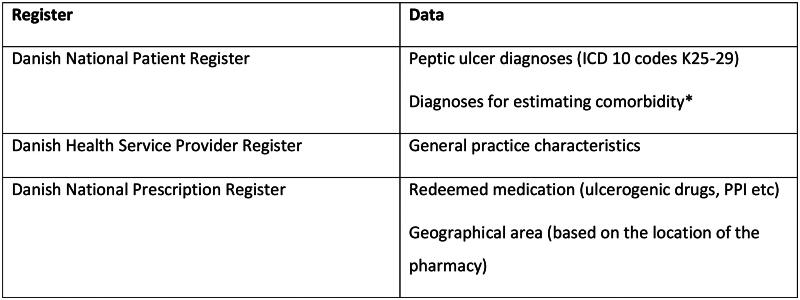
An overview of the registers used in the study and which data we retrieved from each register. *The diagnoses diabetes and COPD were identified as either a diagnose in the Danish National Patient Register or redeeming of a prescription for medication targeting diabetes or COPD as these comorbidities often are diagnosed and treated solely in general practice and therefore not necessarily identifiable in the Danish National Patient Register.

Danish citizens are registered with a unique civil registration number and likewise is each general practice assigned their own identification number. These identification numbers enable accurate linkage of patients and general practices through all national registers [18]. A description of which registers were used for retrieval of relevant variables is given in Figure 1.

### Patient cohort sampling

A risk model to predict the individual risk of aspirin/NSAID related upper gastrointestinal bleeding has been developed combining the patient’s age, sex, prior ulcer and use of other drugs increasing the risk of gastrointestinal hemorrhage to accurately estimate the individual risk of upper gastrointestinal bleeding [5]. In the present study, we used this risk model to identify all Danes who were at risk of peptic ulcer bleeding at the index date 15 January 2019. Ulcerogenic drugs were defined as NSAIDs or aspirin (anatomical therapeutic chemical classification (ATC) codes M01A/B01AC06 + N02BA), and potentially aggravating drugs were defined as anticoagulants (ATC code B01A), systemic corticosteroids (ATC code H02) and selective serotonin reuptake inhibitors (SSRIs) (ATC code N06AB). These drugs are well known to increase the risk of peptic ulcer and/or increase the risk of hemorrhage. Further, the relative risk associated with each drug is available [19,20]. The drugs were considered to be used concomitantly if the patient had redeemed the prescriptions on the index date or prior to redeeming drug number 2 had redeemed a quantity of drug number 1 large enough to be covering the date of redeeming drug number 2. We used the risk model to identify a group of patients at high risk of peptic ulcer bleeding (incidence rate ratio IRR ≥3.5% per year).

### Defining PPI prophylaxis

For patients at high risk of peptic ulcer bleeding, we analyzed the proportion being concomitantly treated with PPIs, ATC code A02BC. The patients were considered to be covered by PPI prophylaxis if they on the index redeemed a prescription for a PPI or previously had redeemed a quantity (number of tablets) of PPI large enough to cover the index date, assuming a dosage of one tablet per day [11].

We retrieved additional data on comedication (as mentioned above) from the Danish National Prescription Register and data on diagnoses from the Danish National Patient Register [21]. Diagnoses included previous peptic ulcer and relevant diagnoses to calculate Charlson Comorbidity Index, providing an estimate of comorbidity *ad modum* Quan [22,23]. Data on age and sex were obtained from sociodemographic registers contained in Statistics Denmark [24,25].

### General practice data

The identified individuals at increased risk of peptic ulcer bleeding were linked to a general practice by identifying the prescriber who was mainly responsible for medications redeemed by the individual the year prior to the index date. As an example, if a patient had redeemed three different medications through the year preceding the index date, the prescriber of at least 2/3 drugs was categorized as the patient’s main prescriber [26].

Further characteristics of the practice were identified through the Danish National Health Service Provider Register. We identified the number of GPs registered at each practice. Practices were defined single-handed practices, if only one established GP was registered, and partnership practice if two or more established GPs were registered. We calculated the number of patients per GP by dividing the practice’s patient list size by the number of established GPs within the practice [17]. In single-handed practices, the GP’s age and sex were retrieved, and in partnership practices we calculated the mean age of the GPs and assessed, whether the GPs were exclusively men or women, predominantly men (>50% men) or predominantly women (>50% women) or equally mixed (50% men/50% women).

The location of all practices was extracted using the municipal location of the pharmacy where the patient predominantly had redeemed prescriptions in the year prior to the index date. Practices within the greater Copenhagen area were categorized as capital area practices. Practices in one of the three largest cities outside Copenhagen were labeled as metropolitan practices and practices in cities with 30,000–100,000 inhabitants were categorized as provincial practices. Towns with <30,000 inhabitants were labeled as either commuter municipalities or rural municipalities depending on the area’s availability of jobs reflecting the area’s economic activity [27].

For each practice, we calculated a ‘PPI coverage proportion’ defined as the proportion of patients at increased risk of peptic ulcer bleeding treated with PPIs within the practice and categorized practices accordingly into five groups. (Proportion of patients at increased risk of peptic ulcer bleeding and treated with PPIs: 0–20%, 21–40%, 41–60%, 61–80% and 81–100%.)

### Statistics

The analyses were conducted both with the entire cohort of general practices and with stratification into single-handed and partnership practices. We did so because the variables age and sex were exact values in single-handed practices, but average values in partnership practices. Mixed effects logistic regression models with patients nested within practice were used to calculate odds ratios (ORs) with 95% confidence intervals (CIs) for associations between PPI use among patients at increased risk of peptic ulcer and practice characteristics. Two regression models were used. Model 1 estimated the crude OR for the association of each practice characteristic and prescribing of PPIs. Model 2 estimated the OR for each practice characteristic, adjusted for both patient characteristics known to be associated with PPI prophylaxis (age, sex and comorbidity) [11] and other practice characteristics included in the analyses.

## Results

**Figure 2.**
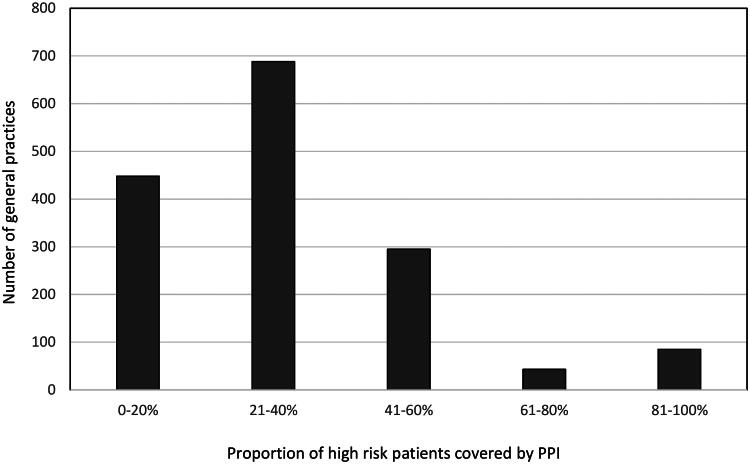
Histogram of the distribution of proportion of high-risk patients covered by PPI among general practices, *N* = 1545.

Among the approximately 1700 general practices in Denmark, we identified 1545 general practices with 55,570 patients at high risk of peptic ulcer bleeding at the index date. Figure 2 demonstrates the proportion of risk patients covered by PPI where 73.5% (1136/1545) of the practices had a PPI coverage proportion of 21–40%. Only in 8.2% (128/1545) of the practices, more than 60% of the high-risk patients were covered by PPI.

Table 1 shows the characteristics of the practices demonstrating an almost even distribution of single-handed and partnership practices. However, the GPs in single-handed practices were substantially older than the GPs in partnership practices.

**Table 1.** Characteristics of general practices, *N* = 1545.

Characteristics	*N* all	*N* single-handed practices	*N* partnership practices
Number of GPs			
1	744	744	–
2	320	–	320
3	218	–	218
4	98	–	98
5	57	–	57
>5	39	–	39
Missing	69	–	–
GP age group			
<45 years	398	81	104
45–49 years	264	85	107
50–54 years	213	90	135
55–59 years	214	105	211
60–64 years	219	177	101
≥65 years	148	206	74
Missing	69	–	–
GP sex			
Men	490	411	79
Predominantly men	146	–	146
Equally mixed	248	–	248
Predominantly women	163	–	163
Women	429	333	96
Missing	69	–	–
GP location			
Capital municipality	478	311	167
Metropolitan municipality	183	80	103
Provincial municipality	310	135	175
Commuter municipality	199	62	137
Rural municipality	306	156	150

Table 2 shows the associations between general practice characteristics and risk patients being coved by prophylactic PPI in terms of crude and adjusted ORs. Practice locations outside the capital area were associated with higher odds of PPI coverage among risk patients. No significant associations were found for GP sex or practice size (Table 2).

**Table 2.** Associations between PPI coverage among patients at increased risk of peptic ulcer bleeding (incidence rate ratio ≥3.5% per year) and general practice characteristics.

Characteristics	Crude OR (95% CI)	Crude *p* value	Adjusted^a^ OR (95% CI)	Adjusted^a^ *p* value
Patients per GP				
<1350	–	–	–	–
1350–1575	0.96 (0.90–1.02)	.173	0.97 (0.91–1.03)	.296
1576–1750	0.96 (0.91–1.02)	.220	0.98 (0.92–1.04)	.569
>1750	0.97 (0.91–1.02)	.232	1.00 (0.94–1.06)	.998
GP location				
Capital municipality	–	–	–	–
Metropolitan municipality	1.32 (1.22–1.43)	.000	1.37 (1.26–1.48)	.000
Provincial municipality	1.13 (1.06–1.20)	.000	1.17 (1.10–1.24)	.000
Commuter municipality	1.19 (1.11–1.27)	.000	1.23 (1.15–1.32)	.000
Rural municipality	1.18 (1.11–1.25)	.000	1.23 (1.16–1.30)	.000
GP sex^b^				
Men	–	–	–	–
Predominantly men	1.06 (0.99–1.13)	.076	1.04 (0.94–1.16)	.418
Equally mixed	1.05 (0.99–1.11)	.123	1.02 (0.94–1.12)	.588
Predominantly women	1.02 (0.96–1.09)	.511	1.01 (0.91–1.12)	.892
Women	1.01 (0.95–1.08)	.797	1.02 (0.95–1.09)	.663
GP age group^b^				
<45 years	–	–	–	–
45–49 years	0.94 (0.87–1.02)	.144	0.95 (0.87–1.02)	.173
50–54 years	1.01 (0.93–1.09)	.866	1.03 (0.95–1.11)	.495
55–59 years	0.95 (0.87–1.02)	.167	0.98 (0.90–1.06)	.552
60–64 years	0.97 (0.90–1.06)	.528	1.02 (0.94–1.11)	.561
≥65 years	1.03 (0.94–1.13)	.469	1.10 (1.00–1.21)	.046
Number of GPs				
1	–	–	–	–
2	1.03 (0.97–1.09)	.373	1.01 (0.93–1.09)	.870
3	1.03 (0.97–1.09)	.394	0.99 (0.89–1.10)	.868
4	1.07 (1.00–1.15)	.060	1.04 (0.93–1.15)	.496
5	1.05 (0.96–1.15)	.262	1.01 (0.89–1.15)	.881
>5	1.10 (1.00–1.22)	.051	1.06 (0.93–1.21)	.383

^a^ Adjustments were made for patient characteristics and other practice characteristics included in the analyses. Patient characteristics adjusted for were age, sex and comorbidity.

^b^ GP sex is stated as exclusively men or women, predominantly men or women or equally mixed. GP age is the mean age of the GPs in partnership practices.

When stratifying practices into single-handed and partnership practices, we found that high risk patients listed with partnership practices with GPs ≥65 years or practices with only female GPs had a higher odds of PPI coverage compared to practices with GPs aged <45 years (OR 1.23 (95% CI 1.07–1.42)) and practices with only male GPs (OR 1.19 (95% CI 1.05–1.35)). There were no associations between age or sex and PPI coverage among singlehanded practices (Tables 3 and 4).

**Table 3.** Crude and adjusted odds ratios (or) with 95 % confidence intervals (CIs) for the association between characteristics of single-handed practices and PPI use among patients at increased risk of peptic ulcer bleeding (incidence rate ratio ≥3.5% per year).

	Crude OR (95% CI)	Crude *p* value	Adjusted^a^ OR (95% CI)	Adjusted^a^ *p* value
Patients per GP				
<1350	–	–	–	–
1350–1575	0.95 (0.84–1.07)	.385	0.97 (0.85–1.09)	.576
1576–1750	0.92 (0.82–1.05)	.213	0.94 (0.83–1.07)	.351
>1750	1.00 (0.89–1.12)	.988	1.02 (0.91–1.15)	.683
GP location				
Capital municipality	–	–	–	–
Metropolitan municipality	1.25 (1.10–1.43)	.001	1.29 (1.13–1.47)	.000
Provincial municipality	1.11 (1.00–1.22)	.046	1.16 (1.05–1.28)	.004
Commuter municipality	1.12 (0.98–1.28)	.105	1.19 (1.04–1.36)	.014
Rural municipality	1.19 (1.09–1.30)	.000	1.26 (1.15–1.38)	.000
GP sex				
Man	–	–	–	–
Woman	0.96 (0.89–1.04)	.348	0.95 (0.88–1.03)	.252
GP age group				
<45 years	–	–	–	–
45–59 years	0.93 (0.78–1.11)	.442	0.96 (0.81–1.15)	.681
50–54 years	0.88 (0.75–1.02)	.097	0.92 (0.79–1.07)	.294
55–59 years	0.94 (0.82–1.09)	.424	0.97 (0.84–1.11)	.657
60–64 years	0.96 (0.84–1.10)	.577	0.99 (0.86–1.13)	.849
≥65 years	1.00 (0.87–1.15)	.987	1.02 (0.89–1.18)	.759

^a^ In the adjusted analyses, adjustments were made for patient characteristics and other practice characteristics included in the analyses. Patient characteristics adjusted for were age, sex and comorbidity.

**Table 4.** Crude and adjusted odds ratios (or) with 95 % confidence intervals (CIs) for the association between characteristics of partnership practices and PPI use among patients at increased risk of peptic ulcer bleeding (incidence rate ratio ≥3.5% per year).

Variable	Crude OR (95% CI)	Crude *p* value	Adjusted OR (95% CI)	Adjusted *p* value
Patients per GP				
<1350	–	–	–	–
1350–1575	0.98 (0.91–1.05)	.489	0.97 (0.91–1.04)	.461
1576–1750	1.00 (0.93–1.08)	.997	1.01 (0.94–1.09)	.760
>1750	0.95 (0.88–1.02)	.166	0.97 (0.90–1.05)	.425
Practice location				
Capital municipalities	–	–	–	–
Metropolitan municipalities	1.33 (1.20–1.47)	.000	1.40 (1.27–1.55)	.000
Provincial municipalities	1.10 (1.02–1.20)	.018	1.17 (1.08–1.27)	.000
Commuter municipalities	1.18 (1.09–1.29)	.000	1.24 (1.14–1.36)	.000
Rural municipalities	1.13 (1.04–1.23)	.003	1.21 (1.12–1.32)	.000
GP sex^a^				
Men	–	–	–	–
Predominantly men	1.09 (0.99–1.21)	.079	1.14 (1.01–1.28)	.033
Equally mixed	1.08 (0.98–1.19)	.114	1.10 (1.00–1.22)	.050
Predominantly women	1.05 (0.95–1.17)	.301	1.10 (0.98–1.24)	.120
Women	1.12 (0.99–1.27)	.074	1.19 (1.05–1.35)	.006
GP age^a^				
<45 years	–	–	–	–
45–49 years	0.94 (0.86–1.03)	.207	0.95 (0.87–1.04)	.236
50–54 years	1.04 (0.95–1.13)	.436	1.06 (0.97–1.15)	.219
55–59 years	0.95 (0.86–1.04)	.282	0.97 (0.88–1.07)	.523
60–64 years	1.00 (0.89–1.12)	.985	1.04 (0.93–1.17)	.444
≥65 years	1.14 (0.99–1.31)	.076	1.23(1.07–1.42)	.005
Number of GPs				
2	–	–	–	–
3	1.00 (0.93–1.07)	.998	0.97 (0.88–1.07)	.560
4	1.04 (0.97–1.12)	.293	1.03 (0.94–1.12)	.547
5	1.03 (0.93–1.13)	.590	1.00 (0.88–1.13)	.951
≥6	1.07 (0.97–1.19)	.170	1.05 (0.93–1.18)	.444

^a^ GP sex is stated as exclusively men or women, predominantly men or women or equally mixed. GP age is the mean age of the GPs in partnership practices.

In the adjusted analyses, adjustments were made for patient characteristics and other practice characteristics included in the analyses. Patient characteristics adjusted for were age, sex and comorbidity.

There were no significant associations between PPI coverage and other practice characteristics such as number of GPs, other age groups, etc.

## Discussion

### Main findings

In this study, we found that in most general practices less than 40% of the patients at increased risk of peptic ulcer bleeding were covered by prophylactic PPI. We found some geographical variation where practice location outside the capital area was associated with higher odds of PPI coverage among risk patients. Partnership practices with GPs with a mean age ≥65 years or with only female GPs were associated with a higher odds of PPI coverage compared to practices with GPs aged <45 years or with only male GPs. Similar associations were not found for singlehanded practices.

### Strengths and limitations

The major strength of our study is that is based on high quality registers covering the entire nation with a low risk of bias or misclassification [28–30]. Although PPIs are available over the counter, the vast majority is purchased by filling a prescription in primary care [31], i.e. nearly all of risk patients taking PPI are identifiable in this study. An important limitation of our study is that we do not know the indication for prescribing PPI, i.e. we cannot know if the GP had prescribed PPI specifically for ulcer prophylaxis or to treat acid related symptoms. This implies a risk of overestimating the intended ulcer prophylaxis coverage if some high-risk patients are prescribed PPI on symptom indication rather than prophylaxis. On the other hand, we cannot identify patients who are prescribed PPIs but fail to fill in the prescription, hence underestimating the GPs’ awareness of PPI coverage. In a Danish study from 2014, 6.9% of the issued PPI prescriptions were unfilled [32]. Further, we cannot know to what extent the GPs are aware of the patients’ increased risk of ulcer bleeding and after shared decision making with the patients intentionally have chosen not to co-prescribe prophylactic PPI. Nevertheless, it is unlikely that this is the reason for 60% of the risk patients not being prescribed PPI in most practices and our findings call for new methods to increase GP awareness of the individual patient’s ulcer bleeding risk.

The national registers are a valuable source of high-quality data and only in 69/1545 (4.4%) of the practices, we were not able to identify the characteristics sex, age group and number of GPs due to missing data (Table 1). There is no reason to believe that the missing data should not be random and thereby should not entail a risk of bias, but we cannot rule out a risk of overlooking possible associations due to the missing data although the number of missing data is low.

It is a limitation that the location of the general practice was assessed using a proxy (the municipal location of the pharmacy where the patient predominantly had redeemed prescriptions in the year prior to the index date). Further, Denmark is a small and rather homogenous country; hence, it is possible that geographical contrasts are more distinct in other countries.

In the registers, there is no distinction between single-handed practices located separately and single-handed practices located at the same address sharing location and possibly also staff indicating a higher degree of collaboration but without co-ownership of the practices. Therefore, the number of partnership practices only reflects the number of co-owned practices, but some single-handed practices might be administered quite comparable to co-owned practices with a high degree of knowledge-sharing, etc. which might affect guideline awareness and adherence.

Another significant limitation to keep in mind when interpreting the results is that the study is a cross-sectional study. When the exposure (general practice characteristics) and outcome (PPI coverage among high-risk patients) are assessed simultaneously, it is not possible to establish cause and effect relationship but merely to assess associations between exposure and outcome.

### Comparison with existing literature

In a Dutch survey of 3213 patients on aspirin and increased ulcer risk, it was reported that 36% of the patients were not concomitantly treated with regular PPI [33]. Another Dutch study found that 60% of the NSAID-users at increased GI risk were not treated with PPI [34]. A Canadian study showed that few patients initiating NSAID treatment were coprescribed gastroprotective agent even with increasing age [35]. These findings are much in line with our results indicating that underprescribing of ulcer prophylaxis is an issue also in other comparable western healthcare systems.

It is possible that the general practices with a high proportion of risk patients being prescribed PPI have a different prescribing pattern and a general high use of PPI. We cannot know to what extent the GPs prescribed PPI intentionally and specifically as prophylaxis or how many risk patients were prescribed PPI for other indications. It has been demonstrated that increasing GP age is associated with initiating long-term PPI treatment [12]. Hence, our finding of increasing GP age being associated with higher odds of PPI coverage among risk patients may reflect differences in PPI prescribing behavior among GP generations.

## Implications and conclusions

We found that in most general practices, less than 40% of the patients at increased risk of peptic ulcer bleeding were covered by prophylactic PPI. Only few practice characteristics were associated with PPI use among patients at increased risk of peptic ulcer bleeding. It may be that other factors unidentifiable in the registers such as participation in continuous medical education activities [36] are associated with PPI prophylaxis. Our findings indicate that interventions to increase PPI prophylaxis in risk patients should not focus on specific GPs but rather all GPs with a low proportion of risk patients covered by PPI.

In the past years, most efforts to rationalize PPI prescribing have focused on reducing overprescribing; however, our findings that less than 40% of the patients at increased risk of peptic ulcer bleeding were covered by prophylactic PPI suggest a need for efforts to reduce underprescribing as well. Dissemination of knowledge about ulcer prophylaxis in continuous medical education activities may be relevant. Further, development and implementation of decision aids estimating the individual patient’s ulcer risk and risk reducing potential of PPI to guide the GP and patient in decision making about prophylactic PPI could be relevant.
